# Understanding Structure and Agency as Commercial Determinants of Health: Comment on "How Neoliberalism Is Shaping the Supply of Unhealthy Commodities and What This Means for NCD Prevention"

**DOI:** 10.15171/ijhpm.2019.127

**Published:** 2019-12-08

**Authors:** Kelley Lee, Eric Crosbie

**Affiliations:** ^1^Faculty of Health Sciences, Simon Fraser University, Burnaby, BC, Canada.; ^2^School of Community Health Sciences, University of Nevada, Reno, NV, USA.

**Keywords:** Commercial Determinants of Health, Non-communicable Diseases, Corporations, Risk Factors, Structure-Agency

## Abstract

The limited success to date, by the public health community, to address the dramatic rise in non-communicable diseases (NCDs) has prompted growing attention to the commercial determinants of health. This has led to a much needed shift in attention, from metabolic and behavioural risk factors, to the production and consumption of health-harming products by the commercial sector. Building on Lencucha and Thow’s analysis of neoliberalism, in shaping the underlying policy environment favouring commercial interests, we argue for fuller engagement with structure and agency interaction when conceptualising, assessing, and identifying public health measures to address the commercial determinants of health.

## Introduction

Since the late 19th century, the public health community has made substantial progress in understanding the causal role of bacteria, viruses, protozoa and other pathogens in communicable disease morbidity and mortality. Commensurate advances in prevention, control and treatment strategies, along with broader socioeconomic changes, have given rise to the so-called epidemiological transition in many societies. Fast forward to the early twenty-first century and we are witnessing a dramatic shift in recorded causes of death, from communicable to non-communicable diseases (NCDs). The latter – led by cardiovascular diseases, diabetes, cancers, and chronic respiratory diseases – now account for approximately 41 million deaths each year (71% of all deaths annually).^1^

While there is growing evidence that infections have a causal role in many chronic conditions,^2^ two-thirds of NCD deaths worldwide are related to tobacco use, alcohol misuse, unhealthy diets and physical inactivity.^1^ Alongside metabolic risk factors (eg, hypertension), therefore, public health efforts to date have focused on addressing these modifiable behavioural risk factors. Success at stemming the global NCD epidemic has so far been limited. As Horton describes, “progress has been inadequate and disappointingly slow….An advocacy strategy based on four diseases and four risk factors seems increasingly out of touch….And so they [public health community] are paralysed. We need a different approach.”^3^

The concept of the commercial determinants of health offers an important alternative perspective, notably by pushing against the notion that NCDs are primarily self-inflicted, and that people must simply be convinced of the error of their unhealthy ways. Broadly defined as “strategies and approaches used by the private sector to promote products and choices that are detrimental to health,”^4^ this emerging body of work shifts attention, from metabolic and behavioural risks, to the activities of the commercial sector. Of particular concern are the “ways corporations exert power” and the need to “align corporate behaviour more closely with the public good.”^5^ Kickbusch et al, for example, identify four channels through which corporations exert influence – marketing, lobbying, corporate social responsibility initiatives, and supply chain management.^4^ In this way, commercial determinants press for an overdue shift in the public health gaze to more effective regulation of health-harming activities by corporations.

As this approach gains more traction, public health efforts would benefit from fuller understanding of the dual importance of structure and agency when addressing commercial determinants. The relative influence of structure and agency is a longstanding debate in social research. In public health, for example, this debate has pervaded discourses concerning certain types of health promotion interventions.^6^ Briefly, agency concerns the individual and collective capacity to make decisions and act independently through free choice. Structure relates to “the framework within which human agency takes place,”^6^ consisting of relationships, social forces, institutions and rules that shape choices. The interplay between agency and structure, and their relative importance for achieving social change, is an ongoing subject of debate. On the commercial determinants of health, it is essential that efforts to advance this promising approach do not limit the shift in focus, from the agency of individuals, to the agency of corporations.

It is in this respect that Lencucha and Thow’s analysis, of the relationship between neoliberalism and NCDs, is an important advance. The authors address the question of “what underlying conditions have shaped a policy environment that is conducive to the influence of commercial interests”^7^ and, in doing so, open up an expanded conversation about the production and consumption of unhealthy products. Alongside transnational corporations, which further their interests through a variety of business and political strategies, Lencucha and Thow argue that the neoliberal paradigm “has conditioned the policy environment in a way that promotes the supply of unhealthy commodities.”^7^ Enshrined in the institutional fabric of contemporary societies, this dominant paradigm “has given rise to existing systems of governance of product environments, and how these systems create structural barriers to the introduction of meaningful policy action to prevent NCDs by fostering healthy product environments.”^7^ The authors are specifically interested in expanding the explanation of policy incoherence, where economic policies may undermine, and yet are given precedence, over public health policies. Drawing on examples from southern Africa, Lencucha and Thow argue that priority may be given to the production of unhealthy commodities, over protection of public health, even when the former’s contributions to economic development are limited. This is explained by “how the neoliberal paradigm has structured the institutional environment in economic, agricultural and other sectors that shapes the supply of unhealthy products.”^7^

## Defining and Regulating “Undue Influence” by Commercial Interests

While Lencucha and Thow shine important light on how “the influence of health-harming industries on public policy is real and deeply problematic for public health,”^7^ what must then be done to effectively curb corporate agency merits further discussion. To deal with conflicts of interest and “outright corruption,”^7^ Article 5.3 of the World Health Organization (WHO) Framework Convention on Tobacco Control is appropriately identified as an important example to follow. For example, lobbying policy-makers, giving gifts or donations, and industry involvement in policy consultations should be restricted. Article 5.3 requires States Parties to prevent industry interference in setting and implementing public health policies. Beyond public health policy, WHO guidelines “encourage” Parties to “implement measures in all branches of government that may have an interest in, or the capacity to, affect public health policies with respect to tobacco control.”^8^ Efforts to improve policy coherence, and achieve a more integrated approach which spans the breadth of government, will be critical if the agency of health-harming industries is to be effectively regulated.

Importantly, the authors rightly describe how the problem of industry influence goes far beyond “state capture.” Evidence from whistleblowers and internal industry documents have revealed political strategies which are far from visible and, in some cases, intentionally concealed from public view. The revolving door between government and industry^9^; the covert funding of junk science by industry^10^; and the undeclared payment of journalists, celebrities and think tanks to frame public discourse^11^ are patterns of behaviour that span many industries including tobacco, alcohol, food and beverage. These obscured, highly effective political strategies need to be better regulated through improved mechanisms of transparency and accountability. This includes appropriate enforcement and penalties for noncompliance in order to be effective.^12^

More challenging, perhaps, is how undue influence is structurally enabled by the way governments have come to define their own *raison d’etre ,* and thus the policy instruments deemed appropriate and desirable. Industry influence is legitimized by a ‘dominant paradigm’ which goes to the heart of political philosophies underpinning liberal democracies. Pluralism, for instance, assumes that the role of political institutions is to mediate among diverse and competing interest groups. The common good, it is argued, is best achieved through market and civil society actors engaging in robust efforts to influence state actors. Pluralists thus accept industry influence as business as usual.^13^ Undue influence is seen as a problem arising from failings in the system of checks and balances, which then allows commercial actors to undermine the separation of powers underpinning liberal democracies. Public health debates about industry interference have yet to stray into deeper levels of political theory debate such as the potential limitations or design flaws in liberal democracies per se.

## Policy Coherence and the Prioritization of Public Health

By offering a fuller explanation of policy incoherence, Lencucha and Thow appear to assume that public health goals merit priority over other policy goals such as economic growth. In reality, politics is about managing unlimited societal demands with limited public resources. Governing is a messy process of tradeoffs, compromises and mediation among many competing, sometimes incompatible, policy goals. For a variety of reasons, sometimes health policies prevail, but there are situations when they should not. Public interest, in other words, is not the same as public health interest. Economic growth, environmental protection, national security or key infrastructure projects might, for example, be prioritised over public health. As described above, undue influence by vested interests can indeed distort this process. But the fact that public health goals are not prioritised does not necessarily mean that this is the case.

## What Creative Ideas Might We Put Forth to Challenge Neoliberal Hegemony?

The dominance of the neoliberal paradigm, as an underlying condition shaping “a policy environment that is conducive to the influence of commercial interests,” is a daunting but essential revelation. Unhealthy products that contribute to NCDs arise as much, if not more so, from structural or “underlying conditions,” as agency or the choices made by consumers, producers and governments. Indeed, some scholars view neoliberalism broadly, as a social ideology and social determinant of health, highlighting the need to incorporate political economy approaches to understanding public health and health inequalities.^14^ How then might one start to change those conditions?

It is first necessary to remind ourselves that neoliberalism is a moment in history, full of contradiction and subject to change. Neoliberalism is presented by Lencucha and Thow as somewhat ahistorical, rather than, playing out over time and place. Even neoliberalism’s most passionate acolytes now recognise that it would be foolish, like Percy Shelley’s *Ozymandias* , to assume the “end of history.” There are deeply troubling warning signs of neoliberalism’s contradictions, most notably, stark wealth inequalities and alarming global climate change, which have generated widespread disillusion with the existing political and economic order.

On unhealthy products, more specifically, mainstream approaches to tackling NCDs have so far overwhelmingly focused on so-called demand-side measures which seek to change the modifiable behaviours of consumers. Yet there are important opportunities to leverage social change by giving attention to the full breadth of global value chains to implement underused supply-side measures (eg, fiscal measures, licensing, taxation). “Climate-smart agriculture measures,” for example, can be broadly categorized into three types of supply-side measures – reducing emissions, enhancing sinks, and fossil fuel offsetting.^15^ Climate change advocates have pressed large institutions worldwide to divest from the fossil fuel industries. Along the journey from leaf to lung, tobacco control advocates have begun to draw attention to extended producer responsibility for the billions of cigarette butts polluting the planet.^16^ Beyond discouraging production and consumption of unhealthy products, in other words, are multiple points of potential public health intervention that can change the underlying structures that shape action.

Finally, there is much work to do within the public health community. Understanding how neoliberal ideas, institutions and interests permeate societies worldwide suggests limited prospects for approaches mainly focused on metabolic and modifiable behavioural risk factors. WHO defines a risk factor as “any attribute, characteristic or exposure of an individual that increases the likelihood of developing a disease or injury.”^17^ This important discussion of the influence of neoliberalism, by Lencucha and Thow, points to an urgent need to better understand the dynamic interaction between structure and agency in creating risk factors for NCDs. This includes understanding of two ideological pillars of neoliberalism – how neoliberalism shapes the structures within which corporations exert their influence (ie, free markets), and how neoliberalism shapes where agency is seen to lie (ie, individual responsibility). Commercial determinants of health thus offer a promising way forward if we go beyond the activities of profit-seeking, health-harming corporations. As a next step, a fuller definition and accompanying metrics, to better define and measure the risks arising from the commercial determinants of health, would significantly enhance explanations of, and actions to address, the alarming rise in NCDs globally.

## Ethical issues

Not applicable.

## Competing interests

Authors declare that they have no competing interests.

## Authors’ contributions

KL and EC conceived the analysis; KL drafted the paper; EC reviewed and provided additional text in response to reviewers’ comments; KL and EC reviewed and approved the final manuscript.

## Authors’ affiliations

^1^Faculty of Health Sciences, Simon Fraser University, Burnaby, BC, Canada. ^2^School of Community Health Sciences, University of Nevada, Reno, NV, USA.
